# The Surgery in President McKinley’s Case

**Published:** 1901-10

**Authors:** John Parmenter

**Affiliations:** Buffalo, N. Y., Professor of anatomy and clinical surgery at the University of Buffalo


### Special Contributions.

### The Surgery in President McKinley's Case.

AN ACCOUNT OF THE OPERATION NARRATED BY ONE OF THE SURGEONS WHO ASSISTED.

By JOHN PARMENTER, M. D., Buffalo, N. Y.,
Professor of anatomy and clinical surgery at the University of Buffalo.

PURSUANT to the request of the editor of the JOURNAL I herewith furnish such data as came within my knowledge in connection with the recent operation upon President McKinley, on September 6, 1901, at the Pan-American Hospital.

The President was shot about ten minutes after four o'clock, and various surgeons were summoned to the spot, I, myself, among the number. On my arrival at the hospital I found Doctors Mann, Mynter, Wasdin and Lee, the latter of St. Louis. I am informed that a consultation had been held, at which it was decided that the President should receive the same general line of treatment which would be indicated and carried out in the case of a person of much less exalted position. In other words, the personality of the patient was not to weigh in the scientific treatment of his case. I was invited by Dr. Mann to participate in the operation, which began as nearly as I can recollect about twenty minutes after five o'clock.

The wounds sustained by the President, as is already known, consisted, first, of an abrasion caused by a glancing ball located near the middle of the sternum and a little to the right. The second bullet entered the abdominal wall, some five or six inches below the left nipple and about two inches to the left of the median line. These figures are approximate but cannot vary much from the exact locations of the external wounds.

An incision was made by Dr. Mann in the long axis of the body some four or five inches in length, the incision dividing the point of entrance of the bullet. On section of the muscles of the abdominal wall it was ascertained that a penetration of the abdominal cavity had occurred. The incision was then lengthened and an examination made of the viscera, when it was revealed that a bullet had entered the stomach an inch or more above the greater curvature, and probably about the middle of the long axis of that viscus. The wound of entrance showed somewhat ragged edges, and through the opening escaped considerable gas from the stomach, as well as a very small quantity of the stomach contents which was quickly caught up by compresses. This wound was carefully sutured by Dr. Mann. The stomach was then drawn farther out of the cavity, Dr. Mann reaching underneath the omentum to get at its posterior wall, where a wound corresponding to that in the anterior wall was found. This was somewhat larger and apparently more ragged than the first one described. It also was carefully sutured. Further search failed to reveal any other lesion in the abdominal cavity or any trace of the bullet. The operation had then lasted somewhat over an hour, and it was deemed inadvisable to prolong the search for the bullet, the belief being that it had passed into the muscles of the back and would there become encysted.

The abdominal cavity was thoroughly irrigated with salt solution two or three times during the operation. The abdominal wound next was sutured, silkworm-gut being used for the retaining sutures, these penetrating all the layers of the abdomen, after which antiseptic dressings were applied.

At the conclusion of the operation the President's pulse was 130, and of fairly good quality. He took the anesthetic throughout the operation without any unpleasant symptoms, due, undoubtedly in large part, to its skilful administration by Dr. Wasdin. Within half an hour the President was transferred in the ambulance to the home of Mr. Milburn.

As my experience with the case and relation to it ended with the President's removal from the hospital, I can give no further data.

519 FRANKLIN STREET.

